# Spectrum Sensing Using Co-Prime Array Based Modulated Wideband Converter

**DOI:** 10.3390/s17051052

**Published:** 2017-05-06

**Authors:** Wanghan Lv, Huali Wang, Shanxiang Mu

**Affiliations:** 1 School of Electronic and Optical Engineering, Nanjing University of Science and Technology, Nanjing 210094, China; 313104002286@njust.edu.cn; 2 College of Communications Engineering, PLA University of Science and Technology, Nanjing 210007, China; wanghl09092009@163.com

**Keywords:** co-prime array, modulated wideband converter, spectrum sensing, minimal sampling rate

## Abstract

As known to us all, it is challenging to monitor wideband signals in frequency domain due to the restriction of hardware. Several practical sampling schemes, such as multicoset sampling and the modulated wideband converter (MWC), have been proposed. In this work, a co-prime array (CA) based modulated wideband converter (MWC) spectrum sensing method is suggested. Our proposed method has the same sampling principle as the MWC but has some advantages compared to MWC. Firstly, CA-based MWC is an array-based MWC system. Each sensor is usually corrupted by independent noise for an array system which can be used for noise averaging, while all channels in conventional MWC have the same receiving noise. Secondly, by incorporating the co-prime array, we can estimate the power spectrum of signal directly employing its second-order statistical properties. Moreover, the system minimal sampling rate can be reduced further because of the reduction of sampling channels. Simulation results show that our method has better performance than traditional methods.

## 1. Introduction

Nowadays, spectral resources traditionally allocated to licensed users by governmental organizations are becoming scant. Cognitive Radio (CR) is an emerging dynamic spectrum management technology which can make the best use of spectral resources in wireless communications [1,2]. How to monitor the spectrum reliably and fast is an essential goal for many scholars [3]. A number of spectrum sensing methods exist, such as filterbank spectrum sensing and multi-taper spectrum sensing, etc. [4]. However, all the above methods are based on Nyquist sampling. In cognitive radio, it is necessary to sense a wide band of spectrum, leading to prohibitively high Nyquist rates. So this will affect the cost and power assumption of high-speed analog-to-digital converters (ADCs). Therefore, it is desirable to design a sub-Nyquist sampling scheme which can effectively estimate the spectrum without loss of any information.

Recently, several sub-Nyquist sampling scheme for spectrum sensing are developed [5,6,7,8]. In [5], the multi-coset sampling (MS) was used, where *m* low-rate cosets were chosen out of *L* cosets of samples. These samples are obtained from time uniformly distributed samples taken at a same sampling in each channel. And there is a different time offset in the sampling origin of each channel. In [6], a synchronous multirate sampling (SMRS) scheme was developed. Unlike the MS method, samples in SMRS are obtained at *m* different sampling rates and the sampling of all channels starts simultaneously at a given time. The above multicoset or time-interleaved approach suffers from some practical issues. Firstly, the radio-frequency (RF) signal is sampled directly. As a result, the signal bandwidth can exceed the analog bandwidth of ADCs. Another practical issue stems from the time shift since it is difficult to maintain accurate delays or synchronization among the ADCs at such high rates. To solve this problem, an analog system, referred to as the modulated wideband converter (MWC) which is comprised of a bank of modulators and low-pass filters is adopted in [7,8,9]. However, conventional MWC systems mainly have two disadvantages. One is that each channel has the same noise, which can’t be used for noise averaging. The other is that we need to choose appropriate periodic functions in different channels. These problems are overcome by adopting a uniform linear array (ULA) based MWC system [10]. In [10], a ULA is used for spectrum sensing where a same periodic function is chosen in each channel. Nevertheless, when signals have high carrier frequencies, there may exist mutual coupling between sensor elements. Moreover, the number of transmissions detected can be no more than the number of physical elements.

In this paper, we propose a spectrum sensing method using an improved MWC system based on co-prime array. The radio frequency (RF) signal impinges on a co-prime array. The received signal in each sensor is multiplied by a same periodic function, low-pass filtered and sampled at a low rate. In the reconstruction process, an enhanced virtual ULA can be produced by vectorizing the data covariance matrix of the co-prime array. It can detect more transmissions than ULA-based MWC or can reduce the system sampling rate further when the number of transmissions is fixed. Besides, each channel in our method is corrupted by different noise, so it has an advantage that the noise can be averaged which will improve sensing performance.

*Notations.* ⊗ and ⊙ denote the Kronecker product and Khatri-rao product, respectively. vec(.) the vectorization operator that turns a matrix into a vector by stacking all columns on top of the another. ∘ denotes the element-wise product. The complex conjugate operator is ${\left(.\right)}^{*}$, the transpose operator is ${\left(.\right)}^{T}$, and the complex conjugate-transpose is ${\left(.\right)}^{H}$. ∠(.) returns the phase of input. eig(.) returns the eigenvalue of input matrix. ⌈.⌉ returns the nearest integer towards positive infinity.

## 2. Array Signal Model

**Figure 1:**
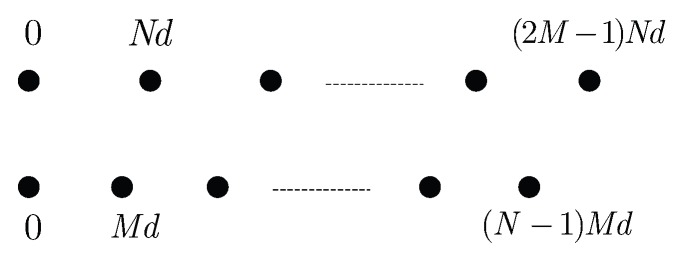
Co-prime array structure.

**Figure 2:**
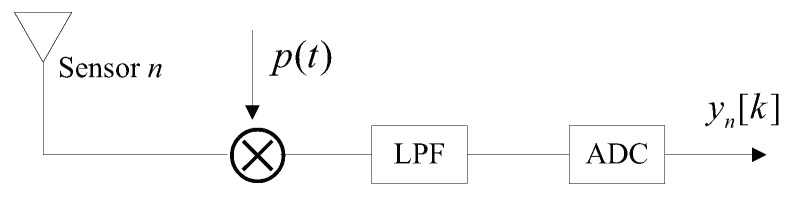
The *n*-th sensor channel structure.

Consider a co-prime linear array consisting of two uniform linear subarrays with 2M and *N* sensor elements where *M* and *N* are co-prime integers and M<N. One has the position set $S_{1}=\left\{Mnd,0\leq n\leq N-1\right\}$ and the other has position set $S_{2}=\left\{Nmd,0\leq m\leq 2M-1\right\}$ where $d=\lambda_{\min}/2$. $\lambda_{\min}$ denotes the wavelength corresponding to the highest frequency of interest. Since these two subarrays share the first sensor, namely the reference sensor, there are N+2M−1 sensors totally. Such array configuration allows most adjacent elements to be spaced farther apart, which is attractive when it is necessary to reduce mutual coupling between sensor elements. Following each sensor includes an analog front-end composed of a mixer with the same periodic function p(t), a low-pass filter and a sampler at rate $f_{s}$. The sensing system is depicted in Figure 1 and Figure 2. In practice, to ensure that all the mixing functions are the same without any distortions. We can first produce the satisfying analog mixing signal p(t), then let it pass through a power divider to obtain several same versions of p(t). Each version of the output of power divider can be used as the mixing function of each channel.

Assume that there are *K* uncorrelated transmissions ${\left\{s_{i}\left(t\right)\right\}}_{i=1}^{K}$ impinging on the array which have identical and known direction of arrival (DOA). Each transmission $s_{i}\left(t\right)$ with the bandwidth $B_{i}$ is modulated by a carrier frequency $f_{i}\in R$. So the received RF signal denoted as z(t) which is bandlimited to $F=\left[-\frac{-f_{Nyq}}{2}\frac{f_{Nyq}}{2}\right]$ can be written as

(1) $$\begin{array}{c} z\left(t\right)=\sum_{i=1}^{K}s_{i}\left(t\right)e^{j2\pi f_{i}t} \end{array}$$

We define $B_{\max}≜\max_{i=1}^{K}B_{i}$ and assume $\min_{i\neq j}\left|f_{i}-f_{j}\right|>B_{\max}$. Then the Fourier transform of z(t) can be written as

(2) $$\begin{array}{c} Z\left(f\right)=\int_{-\infty }^{\infty }z\left(t\right)e^{-j2\pi ft}dt=\sum_{i=1}^{K}S_{i}\left(f-f_{i}\right) \end{array}$$

where $S_{i}\left(f\right)$ is the Fourier transform of $s_{i}\left(t\right)$.

## 3. MWC Based on Co-Prime Array

The co-prime array model is depicted in Section 2. The received signal in each sensor is multiplied by a periodic function p(t) whose period is defined as $T_{p}=1/f_{p}$, low-pass filtered with a filter that has cut-off frequency $f_{s}/2$ and sampled at the low rate $f_{s}$. $T_{s}=1/f_{s}$ is the sampling interval. We define $F_{s}≜\left[-f_{s}/2,f_{s}/2\right]$ and $F_{p}≜\left[-f_{p}/2,f_{p}/2\right]$. Referring to [7], we know that the sampling rate of each channel $f_{s}$ must satisfy $f_{s}\geq f_{p}\geq B_{\max}$, through which all signal information can be reserved in the baseband $F_{s}$ without any loss. Here, for simplicity of analysis, we choose $f_{s}=f_{p}$.

Consider the received signal $z_{n}\left(t\right)$ at the *n*-th sensor of the co-prime array

(3) $$\begin{array}{cc} z_{n}\left(t\right) & =\sum_{i=1}^{K}s_{i}\left(t+\tau_{n}\right)e^{j2\pi f_{i}\left(t+\tau_{n}\right)}\\ & \approx \sum_{i=1}^{K}s_{i}\left(t\right)e^{j2\pi f_{i}\left(t+\tau_{n}\right)} \end{array}$$

where $\tau_{n}=d_{n}\cos\left(\theta \right)/c$ is the time delay for signal arriving at the *n*-th sensor with respect to the reference sensor. θ is measured from the axis which is parallel to the linear array. We will show that θ should not be equal to $90^{\circ }$ because different time delays between different sensors are useful for the estimation of carrier frequencies. $d_{n}\in \left\{S_{1}\cup S_{2}\right\}$ is the position of the *n*-the sensor. *c* is the speed of wave propagation. The approximation in (3) stems from the narrowband assumption on $s_{i}\left(t\right)e^{j2\pi f_{i}t}$. We denote the Fourier transform of $z_{n}\left(t\right)$ as

(4) $$\begin{array}{c} Z_{n}\left(f\right)=\sum_{i=1}^{K}S_{i}\left(f-f_{i}\right)e^{j2\pi f_{i}\tau_{n}} \end{array}$$

**Figure 3:**
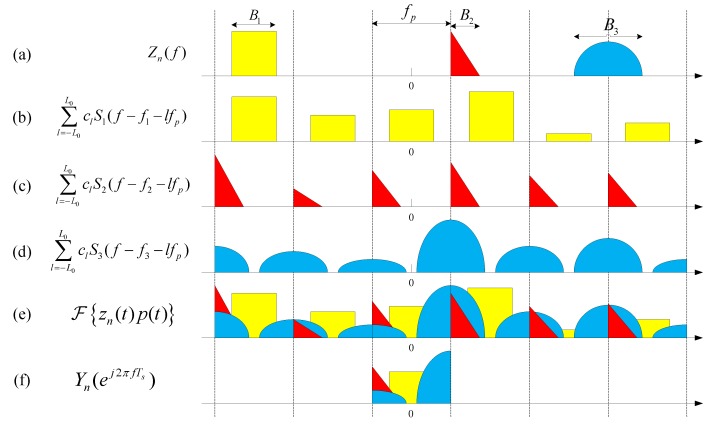
Representations of spectrum at different stages. (**a**) Spectrum of original signal with K=3. (**b**–**d**) are the spectrum of each signal after mixing. (**e**) The spectrum of all signals after mixing. (**f**) The spectrum of all signals after LPF and sampling.

A typical $Z_{n}\left(f\right)$ is shown in Figure 3a. We only show the amplitude spectrum in the figure.

In each sensor channel, it works like one channel of traditional MWC system [7]. So the discrete-time Fourier transform (DTFT) of output $y_{n}\left[k\right]$ in the *n*-th channel can be written as

(5) $$\begin{array}{c} Y_{n}\left(e^{j2\pi fT_{s}}\right)=\sum_{l=-L_{0}}^{L_{0}}c_{l}Z_{n}\left(f-lf_{p}\right),f\in F_{s} \end{array}$$

where

(6) $$\begin{array}{c} c_{l}=\frac{1}{T_{p}}\int_{0}^{T_{p}}p\left(t\right)e^{-j2\pi lt/T_{p}}dt \end{array}$$

and $L_{0}$ is chosen as the smallest integer so that the sum contains all nonzero contributions of $Z_{n}\left(f\right)$ over $F_{s}$. Here, we can choose $L_{0}=\left\lceil \frac{f_{\mathrm{Nyq}}}{2f_{p}}\right\rceil$. For the sake of concreteness, the mixing function p(t) is chosen as a piecewise constant function that alternates between the levels ±1 randomly for each of *P* equal time intervals. Formally,

(7) $$\begin{array}{c} p\left(t\right)=\alpha_{k},\;\frac{kT_{p}}{P}\leq t\leq \frac{\left(k+1\right)T_{p}}{P} \end{array}$$

where 0≤k≤P−1. $\alpha_{k}\in \left\{+1,-1\right\}$. $p\left(t\right)=p\left(t+nT_{p}\right),n\in Z$. Our only request for p(t) is that its Fourier coefficients $c_{l}\neq 0,-L_{0}\leq l\leq L_{0}$.

Substituting (4) into (5), we have

(8) $$\begin{array}{cc} Y_{n}\left(e^{j2\pi fT_{s}}\right) & =\sum_{l=-L_{0}}^{L_{0}}c_{l}\sum_{i=1}^{K}S_{i}\left(f-f_{i}-lf_{p}\right)e^{j2\pi f_{i}\tau_{n}}\\ & =\sum_{i=1}^{K}{\tilde{S}}_{i}\left(f\right)e^{j2\pi f_{i}\tau_{n}},f\in F_{s} \end{array}$$

where ${\tilde{S}}_{i}\left(f\right)≜\sum_{l=-L_{0}}^{L_{0}}c_{l}S_{i}\left(f-f_{i}-lf_{p}\right)$ is a cyclic shifted and scaled version of $S_{i}\left(f\right)$ in the interval $F_{s}$. The whole processing flow in frequency domain is clearly shown in Figure 3.

We write (8) in matrix form as

(9) $$\begin{array}{c} Y\left(e^{j2\pi fT_{s}}\right)=A\tilde{S}\left(f\right),f\in F_{s} \end{array}$$

where $Y\left(e^{j2\pi fT_{s}}\right)={\left[Y_{1}\left(e^{j2\pi fT_{s}}\right),\ldots ,Y_{N+2M-1}\left(e^{j2\pi fT_{s}}\right)\right]}^{T}$ and $\tilde{S}\left(f\right)={\left[{\tilde{S}}_{1}\left(f\right),\ldots ,{\tilde{S}}_{K}\left(f\right)\right]}^{T}$. The matrix A is defined by

(10) $$\begin{array}{c} A=\left(\begin{array}{ccc} e^{j2\pi f_{1}\tau_{1}} & \cdots & e^{j2\pi f_{K}\tau_{1}}\\ \vdots & \ddots & \vdots \\ e^{j2\pi f_{1}\tau_{N+2M-1}} & \cdots & e^{j2\pi f_{K}\tau_{N+2M-1}} \end{array}\right) \end{array}$$

From (9), we can easily get the discretized model under sampling rate $f_{s}$ in the time domain

(11) $$\begin{array}{c} y\left[k\right]=A\tilde{s}\left[k\right] \end{array}$$

## 4. Reconstruction Method

### 4.1. Carrier Frequency Recovery

By invoking (11), the temporal covariance matrix can be written as

(12) $$\begin{array}{ccc} R_{y} & = & E\{y\left[k\right]y^{H}\left[k\right]\}\\ & = & E\{A\tilde{s}\left[k\right]{\tilde{s}}^{H}\left[k\right]A^{H}\}\\ & = & AE\left\{\tilde{s}\left[k\right]{\tilde{s}}^{H}\left[k\right]\right\}A^{H}\\ & = & AR_{\tilde{s}}A^{H} \end{array}$$

Because we assume that the baseband transmissions are uncorrelated with each other, $R_{\tilde{s}}=E\left\{\tilde{s}\left[k\right]{\tilde{s}}^{H}\left[k\right]\right\}$ is a diagonal matrix. Then, by vectorizing the covariance matrix $R_{y}$, we can get

(13) $$\begin{array}{ccc} r_{y} & = & \mathrm{vec}(R_{y})\\ & = & \left(A^{*}⊗A\right)\mathrm{vec}\left(R_{\tilde{s}}\right)\\ & = & \left(A^{*}⊗A\right)\mathrm{diag}\left(R_{\tilde{s}}\right)\\ & = & \left(A^{*}⊙A\right)r_{\tilde{s}} \end{array}$$

where $r_{\tilde{s}}$ is a K×1 vector including the diagonal elements of $R_{\tilde{s}}$. Note that the above equations hold only when all transmissions are uncorrelated with each other. Here, we denote $A_{\mathrm{ca}}≜A^{*}⊙A$ as the manifold matrix of difference coarray. Obviously, the (l,k)-th element of $A_{\mathrm{ca}}$ is given by

(14) $$\begin{array}{ccc} {\left[A_{\mathrm{ca}}\right]}_{l,k} & = & e^{-j2\pi f_{k}d_{n}\cos\left(\theta \right)/c}\cdot e^{j2\pi f_{k}d_{m}\cos\left(\theta \right)/c}\\ & = & e^{j2\pi f_{k}\left(d_{m}-d_{n}\right)\cos\left(\theta \right)/c},\;l=\left(n-1\right)\left(N+2M-1\right)+m \end{array}$$

where $e^{-j2\pi f_{k}d_{n}\cos\left(\theta \right)/c}$ is the (n,k)-th element of $A^{*}$ and $e^{j2\pi f_{k}d_{m}\cos\left(\theta \right)/c}$ is the (m,k)-th element of A. $d_{m},d_{n}\in S_{1}\cup S_{2}$. So we can easily get that the aperture of the difference coarray extends from −(2M−1)Nd to (2M−1)Nd. But this difference coarray is not filled, there are some holes in it. By referring to [11], we know that it has a contiguous set of elements from −(MN+M−1)d to (MN+M−1)d, which acts like a filled virtual uniform linear array (ULA). To make it more clear, we first define the weight function at each element position of the virtual ULA.

**Definition** **1.** *(The weight function, w(l),−(MN+M−1)≤l≤MN+M−1). Consider a co-prime array with its co-prime parameters M and N. Let $S=S_{1}\cup S_{2}$ be the element position set of physical array and D={l|−(MN+M−1)≤l≤MN+M−1} be the element position set of virtual ULA. The weight function is the number of pairs $\left(n,m\right)\in S^{2}$ which have a difference m−n=l, defined as*

(15) $$\begin{array}{ccc} w(l) & = & \mathrm{Cardinality}\;\mathrm{of}\;\mathrm{the}\;\mathrm{set}\;M(l) \end{array}$$

(16) $$\begin{array}{ccc} M(l) & = & \left\{\left(n,m\right)\in S^{2}\;|\;m-n=l\right\} \end{array}$$

For example, if we choose M=2,N=3, then S={0,2,3,4,6,9} and D={l|−7≤l≤7}. The weight function w(l) satisfies w(−7)=1,w(−6)=2, etc.

Based on (13), we denote ${\left[r_{y}\right]}_{\left(n,m\right)}$ as the row of $r_{y}$, which is produced by the *n*-th row of $A^{*}$ and the *m*-th row of A via Khatri-Rao product. So by choosing the continuous lags from −(MN+M−1) to (MN+M−1) and taking the weight function into account, we can get a virtual ULA model

(17) $$\begin{array}{c} r_{y\mathrm{ULA}}=B_{\mathrm{ULA}}r_{\tilde{s}} \end{array}$$

where the *i*-th (i=l+MN+M) element of $r_{y\mathrm{ULA}}$ is

(18) $$\begin{array}{c} {\left[r_{y\mathrm{ULA}}\right]}_{i}=\frac{1}{w(l)}\sum_{\left(n,m)\in M(l\right)}{\left[r_{y}\right]}_{\left(n,m\right)},\;l\in D \end{array}$$

and $B_{\mathrm{ULA}}$, namely the array manifold of the virtual ULA, has the structure

$$\begin{array}{c} \left(\begin{array}{ccc} e^{j2\pi f_{1}{\tilde{\tau }}_{-MN-M+1}} & \cdots & e^{j2\pi f_{K}{\tilde{\tau }}_{-MN-M+1}}\\ \vdots & \ddots & \vdots \\ e^{j2\pi f_{1}{\tilde{\tau }}_{MN+M-1}} & \cdots & e^{j2\pi f_{K}{\tilde{\tau }}_{MN+M-1}} \end{array}\right) \end{array}$$

where ${\tilde{\tau }}_{n}=dn\cos\left(\theta \right)/c,-\left(MN+M-1\right)\leq n\leq MN+M-1$. Based on (17), we have the following sufficient condition for unique solution to ${\left\{f_{i}\right\}}_{i=1}^{K}$. Obviously, $r_{\tilde{s}}$ is equivalent to *K* coherent sources with only one snapshot.

**Theorem** **1.** *Consider a co-prime array consisting of N+2M−1 sensor elements which can be transformed into a virtual filled ULA in (17). If*

(19) $$\begin{array}{c} MN+M>K \end{array}$$

*then (17) has a unique solution of ${\left\{f_{i}\right\}}_{i=1}^{K}$.*

**Proof.**  Because $r_{\tilde{s}}$ is equivalent to *K* coherent sources, we have $\dim(\mathrm{span}\left(r_{\tilde{s}}\right))=1$. And the virtual array acts as a filled ULA with inter-element spacing *d* satisfies $d\leq \frac{c}{2f_{\mathrm{Nyq}}}$ and the number of virtual elements is 2MN+2M−1. So we refer the reader to [10] which deals with the physical ULA case. With these substitutions, the result follows from Theorem 1 in [10]. ☐

Next, we define

(20) $$\begin{array}{ccc} R_{y\mathrm{ULA}} & = & r_{y\mathrm{ULA}}r_{y\mathrm{ULA}}^{H}\\ & = & B_{\mathrm{ULA}}r_{\tilde{s}}r_{\tilde{s}}^{H}B_{\mathrm{ULA}}^{H}\\ & = & B_{\mathrm{ULA}}R_{\tilde{s}\tilde{s}}B_{\mathrm{ULA}}^{H} \end{array}$$

Because

(21) $$\begin{array}{c} \mathrm{rank}\left(R_{y\mathrm{ULA}}\right)=\mathrm{rank}\left(R_{\tilde{s}\tilde{s}}\right)=1 \end{array}$$

we need to implement a spatial smoothing step to enhance the rank of the covariance matrix. As analyzed above, the virtual ULA has the element position from −(MN+M−1)d to (MN+M−1)d. Now, we divide this virtual array into MN+M overlapping subarrays, each with MN+M elements. The *i*-th subarray has sensors located at

(22) $$\begin{array}{c} \left\{(-i+1+n)d,n=0,1,\ldots ,MN+M-1\right\} \end{array}$$

which corresponds to the (MN+M+1−i)-th to (2MN+2M−i)-th rows of $r_{y\mathrm{ULA}}$. So we have

(23) $$\begin{array}{c} r_{y\mathrm{ULA}i}=B_{\mathrm{ULA}i}r_{\tilde{s}} \end{array}$$

where $B_{\mathrm{ULA}i}$ is a (MN+M)×K matrix consisting of the (MN+M+1−i)-th to (2MN+2M−i)-th rows of $B_{\mathrm{ULA}}$ which has the structure

(24) $$\begin{array}{c} B_{\mathrm{ULA}i}=\left(\begin{array}{ccc} e^{j2\pi f_{1}\left(1-i\right)d\cos\left(\theta \right)/c} & \cdots & e^{j2\pi f_{K}\left(1-i\right)d\cos\left(\theta \right)/c}\\ \vdots & \ddots & \vdots \\ e^{j2\pi f_{1}\left(MN+M-i\right)d\cos\left(\theta \right)/c} & \cdots & e^{j2\pi f_{K}\left(MN+M-i\right)d\cos\left(\theta \right)/c} \end{array}\right) \end{array}$$

Obviously, from the above structure, we can get

(25) $$\begin{array}{c} B_{\mathrm{ULA}i}=B_{\mathrm{ULA}1}\Phi^{i-1} \end{array}$$

where Φ is a diagonal matrix with its diagonal elements as $\left\{e^{-j2\pi f_{1}d\cos\left(\theta \right)/c},\ldots ,e^{-j2\pi f_{K}d\cos\left(\theta \right)/c}\right\}$. So, we rewrite (23) as

(26) $$\begin{array}{c} r_{y\mathrm{ULA}i}=B_{\mathrm{ULA}1}\Phi^{i-1}r_{\tilde{s}} \end{array}$$

Then, we can get the spatially smoothed matrix

(27) $$\begin{array}{ccc} R_{\mathrm{ss}} & = & \frac{1}{MN+M}\sum_{i=1}^{MN+M}R_{y\mathrm{ULA}i} \end{array}$$

where

(28) $$\begin{array}{ccc} R_{y\mathrm{ULA}i} & = & r_{y\mathrm{ULA}i}r_{y\mathrm{ULA}i}^{H}\\ & = & B_{\mathrm{ULA}1}\Phi^{i-1}r_{\tilde{s}}r_{\tilde{s}}^{H}{\left(\Phi^{i-1}\right)}^{H}B_{\mathrm{ULA}1}^{H} \end{array}$$

The spatially smoothed matrix $R_{\mathrm{ss}}$ can be used to estimate carrier frequencies by the following theorem.

**Theorem** **2.** *Consider the spatially smoothed matrix $R_{\mathrm{ss}}$ in (27) and define a K×K diagonal matrix $\Lambda_{s}$ with its diagonal elements as the covariances of K targets. Then, we have*

(29) $$\begin{array}{ccc} R_{\mathrm{ss}} & = & {\tilde{R}}^{2}\\ \tilde{R} & = & \frac{1}{\sqrt{MN+M}}B_{\mathrm{ULA}1}\Lambda B_{\mathrm{ULA}1}^{H} \end{array}$$

**Proof.**  The proof follows the same lines as Theorem 1 in [12], only substituting the values of $B_{\mathrm{ULA}1}$ and $\Lambda_{s}$ in our paper. ☐

By decomposing $\tilde{R}$ using the singular value decomposition, we have

(30) $$\begin{array}{c} \tilde{R}=\left[U_{1}\;U_{2}\right]\left[\begin{array}{cc} \Lambda_{s} & 0\\ 0 & 0 \end{array}\right]V^{H} \end{array}$$

The columns of the matrix $\left[U_{1}\;U_{2}\right]$ are the left singular vectors of $\tilde{R}$, where $U_{1}$ contains the vectors corresponding to the first *K* singular values, $\Lambda_{s}$ is a K×K diagonal matrix with the *K* first singular values of $\tilde{R}$, and V contains the right singular vector of $\tilde{R}$. Based on (29) and (30), we know that there exists an invertible K×K matrix T such that

(31) $$\begin{array}{c} U_{1}=B_{\mathrm{ULA}1}T \end{array}$$

Consider the first MN+M−1 rows of $U_{1}$, we have

(32) $$\begin{array}{c} U_{11}=B_{\mathrm{ULA}11}T \end{array}$$

Similarly, we can have the last MN+M−1 rows of $U_{1}$

(33) $$\begin{array}{c} U_{12}=B_{\mathrm{ULA}12}T \end{array}$$

where $B_{\mathrm{ULA}11}$ is the virtual sub-array consisting of element positions {0,d,…,(MN+M−2)d} and $B_{\mathrm{ULA}12}$ is the virtual sub-array consisting of elements positions {d,2d,…,(MN+M−1)d}. So, we can get the relationship between $B_{\mathrm{ULA}11}$ and $B_{\mathrm{ULA}12}$ as

(34) $$\begin{array}{c} B_{\mathrm{ULA}11}=B_{\mathrm{ULA}12}\Phi \end{array}$$

where Φ is a diagonal matrix which is defined in (26). So we rewrite (32) as

(35) $$\begin{array}{c} U_{11}=B_{\mathrm{ULA}12}\Phi T \end{array}$$

Here, we use the least squares recovery

(36) $$\begin{array}{c} \Psi =U_{12}^{†}U_{11} \end{array}$$

Then, we have

(37) $$\begin{array}{c} {\hat{f}}_{i}=∠\left(\mathrm{eig}\left(\Psi_{i,i}\right)\right)\frac{c}{2\pi d\cos(\theta )} \end{array}$$

where $\Psi_{i,i}$ is the *i*-th diagonal element of Ψ.

**Remark** **1.** *It can be seen from (37) that θ can not be equal to $90^{\circ }$. And the performance of carrier frequency estimation is affected by θ. Because cos(θ) is the denominator term in (37), a small cos(θ) will amplify the error which is caused by the calculation of* **Ψ**. *Assuming $\theta \in \left[0,90^{\circ }\right)\cup \left(90^{\circ },180^{\circ }\right]$, the closer to $0^{\circ }$ or $180^{\circ }$ the impinging direction θ is, the smaller the estimation error is. Conversely, the closer to $90^{\circ }$, the larger the error is. In practice, if we know that θ is approaching $90^{\circ }$, we can add an adjustable known time delay line after each sensor which is equivalent to rotating the array with a known angle. If we denote the man-made time delay as $d_{n}\cos\left(\theta_{a}\right)/c$, then the denominator term in (37) is modified as $\cos\left(\theta \right)+\cos\left(\theta_{a}\right)$. In the following discussion, we consider the case that θ is close to $0^{\circ }$ or $180^{\circ }$ for simplicity.*

### 4.2. Signal Power Spectrum Recovery

Once the carrier frequencies are recovered, the steering matrix defined in (17) can be constructed. So in this subsection, we will first consider the power spectrum recovery of $\tilde{s}\left[k\right]$. After that, we will investigate how to recover the power spectrum of s[k] from $\tilde{s}\left[k\right]$.

By invoking (9), we consider the signal model in the frequency domain. Define the autocorrelation matrix of $Y(e^{j2\pi fT_{s}})$ as $R_{Y}\left(e^{j2\pi fT_{s}}\right)≜E\left\{Y\left(e^{j2\pi fT_{s}}\right)Y^{H}\left(e^{j2\pi fT_{s}}\right)\right\}$. Similarly, define $R_{\tilde{S}}\left(f\right)≜E\left\{\tilde{S}\left(f\right){\tilde{S}}^{H}\left(f\right)\right\}$ and $R_{S}\left(f\right)≜E\left\{S\left(f\right)S^{H}\left(f\right)\right\}$ for $f\in F_{s}$. Then, we have

(38) $$\begin{array}{c} R_{Y}\left(e^{j2\pi fT_{s}}\right)=AR_{\tilde{S}}\left(f\right)A^{H},f\in F_{s} \end{array}$$

Due to the assumption that all transmissions are uncorrelated with each other, so $R_{\tilde{S}}\left(f\right),f\in F_{s}$ and $R_{S}\left(f\right),f\in F_{s}$ are both diagonal matrixes. Then, similar to the processing steps in (13) and (17), by vectorization, removing the redundancies and choosing the continuous lags, we can get the virtual array model in the frequency domain

(39) $$\begin{array}{c} r_{Y\mathrm{ULA}}\left(f\right)=B_{\mathrm{ULA}}r_{\tilde{S}}\left(f\right),f\in F_{s} \end{array}$$

where $r_{\tilde{S}}\left(f\right)$ is a K×1 vector which contains the diagonal elements of $R_{\tilde{S}}\left(f\right)$. Similarly, we denote $r_{S}\left(f\right)$ as a K×1 vector which contains the diagonal elements of $R_{S}\left(f\right)$. From (17), $B_{\mathrm{ULA}}$ is a Vandermonde matrix, it has full column rank if and only if (2MN+2M−1)≥K. Referring to Theorem 1, if the sufficient condition (19) is satisfied, $B_{\mathrm{ULA}}$ will have full column rank. Then we can obtain the power spectrum of $\tilde{S}\left(f\right)$ by inverting the steering matrix,

(40) $$\begin{array}{c} r_{\tilde{S}}\left(f\right)=B_{\mathrm{ULA}}^{†}r_{Y\mathrm{ULA}}\left(f\right),f\in F_{s} \end{array}$$

As analyzed in the third section, $\tilde{S}\left(f\right)$ is a cyclic and shifted version of S(f).

Consider the *i*-th transmission $S_{i}\left(f^{\prime }\right),f^{\prime }\in F_{p}$. It holds that

(41) $$\begin{array}{c} {\tilde{S}}_{i}\left(f^{\prime }\right)=c_{l_{a}}S_{i}\left(f^{\prime }-f_{i}-l_{a}f_{p}\right), \end{array}$$

where $l_{a}$ is known as

(42) $$\begin{array}{c} l_{a}=\left\lfloor \frac{f_{i}+f^{\prime }+f_{p}/2}{f_{p}}\right\rfloor \end{array}$$

Then we have the relationship between the power spectrum of $S_{i}\left(f^{\prime }\right)$ and ${\tilde{S}}_{i}\left(f^{\prime }\right)$,

(43) $$\begin{array}{ccc} {\left[r_{\tilde{S}}\left(f^{\prime }\right)\right]}_{i} & ≜ & E\{{\tilde{S}}_{i}\left(f^{\prime }\right){\tilde{S}}_{i}^{*}\left(f^{\prime }\right)\}\\ & = & E\{c_{l_{a}}S_{i}\left(f^{\prime }-f_{i}-l_{a}f_{p}\right)S_{i}^{*}\left(f^{\prime }-f_{i}-l_{a}f_{p}\right)c_{l_{a}}^{*}\}\\ & = & |c_{l_{a}}{|}^{2}E\left\{S_{i}\left(f^{\prime }-f_{i}-l_{a}f_{p}\right)S_{i}^{*}\left(f^{\prime }-f_{i}-l_{a}f_{p}\right)\right\}\\ & = & |c_{l_{a}}{|}^{2}{\left[r_{S}\left(f^{\prime }-f_{i}-l_{a}f_{p}\right)\right]}_{i} \end{array}$$

where ${\left[r_{\tilde{S}}\left(f^{\prime }\right)\right]}_{i}$ is the *i*-th element of the K×1 vector $r_{\tilde{S}}\left(f^{\prime }\right)$ and ${\left[r_{S}\left(f^{\prime }-f_{i}-l_{a}f_{p}\right)\right]}_{i}$ is the the *i*-th element of the K×1 vector $r_{S}\left(f^{\prime }-f_{i}-l_{a}f_{p}\right)$. After a change of variables,

(44) $$\begin{array}{c} {\left[r_{S}\left(f^{\prime }\right)\right]}_{i}=\frac{1}{|c_{l_{a}}{|}^{2}}{\left[r_{\tilde{S}}\left(f^{\prime }-f_{i}-l_{a}f_{p}\right)\right]}_{i},f^{\prime }\in F_{p} \end{array}$$

Observing (43) and (44), the equality in (44) holds if and only if $c_{l_{a}}\neq 0$.

### 4.3. Comparison with Previous MWC Systems

By referring to conventional MWC [7] and ULA-based MWC [10], we can have the following conclusions. Firstly, we compare our proposed CA-based MWC with ULA-based MWC and conventional MWC. Our method processes the signal in the co-array domain, while the latter two methods process signal in physical sensor (channel) domain. That means, if we fix the number of physical sensors or channels as N+2M−1, then our proposed CA-based method can produce a virtual ULA which has MN+M elements. It is much larger than that of ULA-based MWC and conventional MWC which can increase the system’s robustness to noise. Another difference is that our method can directly recover the power spectrum of impinging signal, while the latter methods must first recover the signal itself after which the power spectrum is calculated. Here, we need to point out a disadvantage as shown in (13) that the impinging signal for our method must be uncorrelated with each other. Secondly, we compare CA-based MWC, ULA-based MWC with conventional MWC. In our proposed CA-based MWC and ULA-based MWC, carrier frequencies are first estimated, then the baseband transmissions are estimated. For conventional MWC, there’s no need to estimate carrier frequencies, all RF signals are estimated directly. In addition, each channel of CA-based MWC and ULA-based MWC is corrupted by independent noise, while each channel of conventional MWC is corrupted by the same noise. Lastly, we compare our method with ULA-based MWC. Besides a difference about the number of sensors, another difference is that our proposed system is a sparse array system while ULA-based MWC is a filled array system. As we all know, the closer the sensors are, the more correlated their samples are, which can affect the performance. The differences among these three methods are shown clearly in Table 1 where × denotes “Not exist”.

## 5. Robustness Analysis for Imperfect p(t) Among Different Channels

Because the mixing function p(t) in each channel is produced by a power divider, there may be amplitude and phase imbalances of p(t) among different channels. In this section, we will analyze this issue.

Here, we denote the amplitude and phase imbalances of the *n*-th (n=1,…,N+2M−1) channel as $\delta_{n}$ and $\varphi_{n}$, respectively. We assume that the amplitude imbalances ${\left\{\delta_{n}\right\}}_{n=1}^{N+2M-1}$ are i. i. d random variables with Gaussian distribution $N(0,\sigma_{\delta }^{2})$ and the phase imbalances ${\left\{\varphi_{n}\right\}}_{n=1}^{N+2M-1}$ are i. i. d random variables distributed uniformly in [−ρ/2,ρ/2]. Moreover, ${\left\{\varphi_{n}\right\}}_{n=1}^{N+2M-1}$ and ${\left\{\delta_{n}\right\}}_{n=1}^{N+2M-1}$ are independent with each other. So, for the *n*-th channel, the imperfect mixing function $p_{n}\left(t\right)$ can be modeled as

(45) $$\begin{array}{c} p_{n}\left(t\right)=\left(1+\delta_{n}\right)e^{-j\varphi_{n}}p\left(t\right) \end{array}$$

where p(t) is the ideal mixing function. We denote the Fourier coefficients of $p_{n}\left(t\right)$ as ${\tilde{c}}_{n,l}$. Then, we have

(46) $$\begin{array}{ccc} {\tilde{c}}_{n,l} & = & \frac{1}{T_{p}}\int_{0}^{T_{p}}p_{n}\left(t\right)e^{-j2\pi lt/T_{p}}dt\\ & = & \frac{1}{T_{p}}\int_{0}^{T_{p}}\left(1+\delta_{n}\right)e^{-j\varphi_{n}}p\left(t\right)e^{-j2\pi lt/T_{p}}dt\\ & = & \left(1+\delta_{n}\right)e^{-j\varphi_{n}}c_{l} \end{array}$$

where $c_{l}$ is the ideal Fourier coefficients of p(t). Based on (8), we can obtain the perturbed model

(47) $$\begin{array}{ccc} {\hat{Y}}_{n}\left(e^{j2\pi fT_{s}}\right) & = & \sum_{l=-L_{0}}^{L_{0}}\left(1+\delta_{n}\right)e^{-j\varphi_{n}}c_{l}\sum_{i=1}^{K}S_{i}\left(f-f_{i}-lf_{p}\right)e^{j2\pi f_{i}\tau_{n}}\\ & = & \sum_{i=1}^{K}{\tilde{S}}_{i}\left(f\right)e^{j2\pi f_{i}\tau_{n}}\left(1+\delta_{n}\right)e^{-j\varphi_{n}},f\in F_{s} \end{array}$$

We write (47) in matrix form as

(48) $$\begin{array}{c} \hat{Y}\left(e^{j2\pi fT_{s}}\right)=\sum_{i=1}^{K}\left(a_{i}\circ p\right){\tilde{S}}_{i}\left(f\right),f\in F_{s} \end{array}$$

where $a_{i}$ is the *i*-th column of A defined in (10) and $p={\left[\left(1+\delta_{1}\right)e^{-j\varphi_{1}},\ldots ,\left(1+\delta_{N+2M-1}\right)e^{-j\varphi_{N+2M-1}}\right]}^{T}$. Then, we can easily get the temporal perturbed model under sampling rate $f_{s}$ as

(49) $$\begin{array}{c} \hat{y}\left[k\right]=\sum_{i=1}^{K}\left(a_{i}\circ p\right){\tilde{s}}_{i}\left[k\right] \end{array}$$

Now we consider the covariance matrix ${\hat{R}}_{y}=E\left\{\hat{y}\left[k\right]{\hat{y}}^{H}\left[k\right]\right\}$. The structure of ${\hat{R}}_{y}$ is given by the following corollary.

**Theorem** **3.** *In the CA-based MWC system, if we consider the amplitude and phase imbalances for mixing function p(t) among different channels, then the perturbed covariance matrix is given by*

(50) $$\begin{array}{ccc} {\hat{R}}_{y} & = & \sum_{i=1}^{K}{\left[r_{\tilde{s}}\right]}_{i}\left(a_{i}a_{i}^{H}\right)\circ E\\ & = & R_{y}\circ E \end{array}$$

*where ${\left[r_{\tilde{s}}\right]}_{i}$ is the i-th element of $r_{\tilde{s}}$ defined in (13). E is a matrix with $1+\sigma_{\delta }^{2}$ on its diagonal and ${\mathrm{sinc}}^{2}\left(\rho /2\right)$ elsewhere.*

**Proof.**  The perturbed covariance matrix can be obtained by

(51) $$\begin{array}{ccc} {\hat{R}}_{y} & = & E\{\hat{y}\left[k\right]{\hat{y}}^{H}\left[k\right]\}\\ & = & E\left\{\sum_{i=1}^{K}\left(a_{i}\circ p\right)\left(a_{i}^{H}\circ p^{H}\right){\left|{\tilde{s}}_{i}\left[k\right]\right|}^{2}\right\}\\ & = & E\left\{\sum_{i=1}^{K}\left(a_{i}a_{i}^{H}\right)\circ \left(pp^{H}\right){\left|{\tilde{s}}_{i}\left[k\right]\right|}^{2}\right\}\\ & = & \sum_{i=1}^{K}\left(a_{i}a_{i}^{H}\right)\circ E\left\{(pp^{H})\right\}{\left[r_{\tilde{s}}\right]}_{i} \end{array}$$

We denote $P=pp^{H}$, our task is to find the expectation of P. We first investigate the distribution of diagonal elements of P. We have

(52) $$\begin{array}{ccc} E\{{\left[P\right]}_{n,n}\} & = & E\{\left(1+\delta_{n}\right)e^{-j\varphi_{n}}{\left(1+\delta_{n}\right)}^{*}e^{j\varphi_{n}}\}\\ & = & E\{{\left(1+\delta_{n}\right)}^{2}\}\\ & = & E\{1+\delta_{n}^{2}+2\delta_{n}\}\\ & = & 1+\sigma_{\delta }^{2},\;n=1,\ldots ,N+2M-1 \end{array}$$

For the off-diagonal elements of P, we have

(53) $$\begin{array}{ccc} E\{{\left[P\right]}_{n,m}\} & = & E\{\left(1+\delta_{n}\right)e^{-j\varphi_{n}}{\left(1+\delta_{m}\right)}^{*}e^{j\varphi_{m}}\}\\ & = & E\{\left(1+\delta_{n}\right){\left(1+\delta_{m}\right)}^{*}e^{j(\varphi_{m}-\varphi_{n})}\}\\ & = & E\{e^{j(\varphi_{m}-\varphi_{n})}\}\\ & = & E\{e^{j\beta_{mn}}\},n,m=1,\ldots ,N+2M-1,\;m\neq n \end{array}$$

where $\beta_{mn}≜\varphi_{m}-\varphi_{n}$, namely the difference of two independent random variables with uniform distribution in [−ρ/2,ρ/2]. So we can easily obtain that the probability density function (pdf) of $\beta_{mn}$ is a triangular function in the interval [−ρ,ρ]:

(54) $$f_{\beta_{mn}}\left(\beta \right)=\{\begin{array}{cc} \frac{\rho +\beta }{\rho^{2}}, & -\rho \leq \beta <0\\ \frac{\rho -\beta }{\rho^{2}}, & 0\leq \beta \leq \rho \end{array}$$

By integration, the expectation of ${\left[P\right]}_{n,m}$ is calculated as

(55) $$\begin{array}{ccc} E\{{\left[P\right]}_{n,m}\} & = & E\{e^{j\beta_{mn}}\}\\ & = & \int_{-\rho }^{\rho }e^{j\beta }f_{\beta_{mn}}\left(\beta \right)d\beta \\ & = & {\left(\frac{\sin(\rho /2)}{\rho /2}\right)}^{2}\\ & = & {\mathrm{sinc}}^{2}\left(\rho /2\right) \end{array}$$

In (50), E=E{P} which completes the proof. ☐

Using Theorem 3, we can know that the structured noise caused by the amplitude and phase imbalances of p(t) can be written as

(56) $$\begin{array}{ccc} R_{y}-{\hat{R}}_{y} & = & \sum_{i=1}^{K}{\left[r_{\tilde{s}}\right]}_{i}\left(a_{i}a_{i}^{H}\right)\circ \left(1-E\right)\\ & = & R_{y}\circ \left(1-E\right) \end{array}$$

where 1 is an all-ones matrix with the size (N+2M−1)×(N+2M−1). Obviously, the structured noise acts like the additive colored correlated noise which can degrade the final estimation performance.

**Corollary** **1.** *Let ρ≪1, then the deviation of perturbed covariance matrix from the ideal covariance matrix is given by*

(57) $$\begin{array}{c} {∥R_{y}-{\hat{R}}_{y}∥}_{F}={∥r_{\tilde{s}}∥}_{2}\sqrt{\left(N+2M-1\right)[\sigma_{\delta }^{4}+\left(N+2M-2\right)\rho^{4}/144]} \end{array}$$

*where ${∥.∥}_{F}$ is the Frobenius norm.*

**Proof.**  Obviously, the *n*-th diagonal element of $R_{y}-{\hat{R}}_{y}$ have the following equation

(58) $$\begin{array}{ccc} {\left|{\left(R_{y}-{\hat{R}}_{y}\right)}_{n,n}\right|}^{2} & = & \sum_{i=1}^{K}{\left|{\left[r_{\tilde{s}}\right]}_{i}\right|}^{2}\sigma_{\delta }^{4}\\ & = & \sigma_{\delta }^{4}{∥r_{\tilde{s}}∥}_{2}^{2} \end{array}$$

For the (n,m)-th off-diagonal element of $R_{y}-{\hat{R}}_{y}$, we have

(59) $$\begin{array}{ccc} {\left|{\left(R_{y}-{\hat{R}}_{y}\right)}_{n,m}\right|}^{2} & = & \sum_{i=1}^{K}{\left|{\left[r_{\tilde{s}}\right]}_{i}\right|}^{2}{\left(1-{\mathrm{sinc}}^{2}\left(\rho /2\right)\right)}^{2}\\ & = & {\left(1-{\mathrm{sinc}}^{2}\left(\rho /2\right)\right)}^{2}{∥r_{\tilde{s}}∥}_{2}^{2} \end{array}$$

Then, we can easily get

(60) $$\begin{array}{ccc} {∥R_{y}-{\hat{R}}_{y}∥}_{F} & = & ∥r_{\tilde{s}}{∥}_{2}\sqrt{\left(N+2M-1\right)\sigma_{\delta }^{4}+\left(N+2M-2\right)\left(N+2M-1\right){\left(1-{\mathrm{sinc}}^{2}\left(\rho /2\right)\right)}^{2}}\\ & = & ∥r_{\tilde{s}}{∥}_{2}\sqrt{\left(N+2M-1\right)[\sigma_{\delta }^{4}+\left(N+2M-2\right){\left(1-{\mathrm{sinc}}^{2}\left(\rho /2\right)\right)}^{2}]} \end{array}$$

Due to the assumption that ρ≪1, so $1-{\mathrm{sinc}}^{2}\left(\rho /2\right)$ in (60) can be approximated as $\rho^{2}/12$. Then we can obtain (57). ☐

## 6. Choice of Co-Prime Parameters

In Theorem 1, we know how to choose co-prime parameters *M* and *N* to satisfy the unique recovery condition. In application, the number of total sensors is usually fixed, namely N+2M−1=Q. As analyzed in Section 3, a virtual array whose elements are given by the difference coarray can be produced by vectorizing the data covariance matrix. Although the coarray of co-prime array has some missing elements or ’holes’, we can only employ that part of the coarray which has the continuous elements from −(MN+M−1) to MN+M−1. So it acts like a filled ULA which has 2MN+2M−1 elements. If we can increase the aperture of the virtual ULA, then the number of transmissions which can be detected also increases. So we have the following optimization

(61) $$\begin{array}{c} \max_{M,N}\;2MN+2M-1\\ s.t.\;2M+N-1=Q,M<N\\ \gcd(M,N)=1,M>0,N>0 \end{array}$$

where gcd(.) is a function which returns the greatest common divisor. According to Cauchy inequality [13], we have the optimal solutions $M^{*}$ and $N^{*}$ satisfying $2M^{*}=N^{*}+1=\left(Q+2\right)/2$, namely $M^{*}=\left(Q+2\right)/4$ and $N^{*}=Q/2$ without considering the constrictions. Taking that *M* and *N* are co-prime integers into account, we can search the satisfying optimal solution around $\left(M^{*},N^{*}\right)$.

## 7. Numerical Results

In this section, we assume $f_{\mathrm{Nyq}}=10\;\mathrm{GHz}$, $\theta =0^{\circ }$ and $B_{\max}=50\;\mathrm{MHz}$. In traditional MWC system, because the signal in each sampling channel comes from the same sensor, we can assume that all sampling channels are corrupted by the same additive Gaussian white noise. However, in our proposed array-based MWC system, we can assume that each sampling channel has uncorrelated Gaussian white noise because we use different sensors to receive the signal in different sampling channels. For simplicity, we choose $f_{s}\;=\;f_{p}\;=\;1.3B_{\max}$ in all simulations. The Matlab codes of conventional MWC system can be referred to [14].

### 7.1. Detection Performance

**Figure 4:**
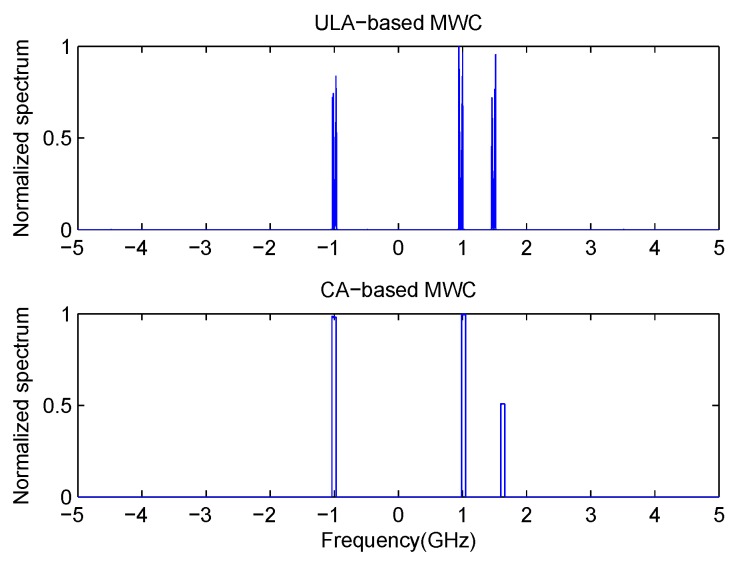
Detection performance in case of 3 transmissions with SNR = 10 dB.

**Figure 5:**
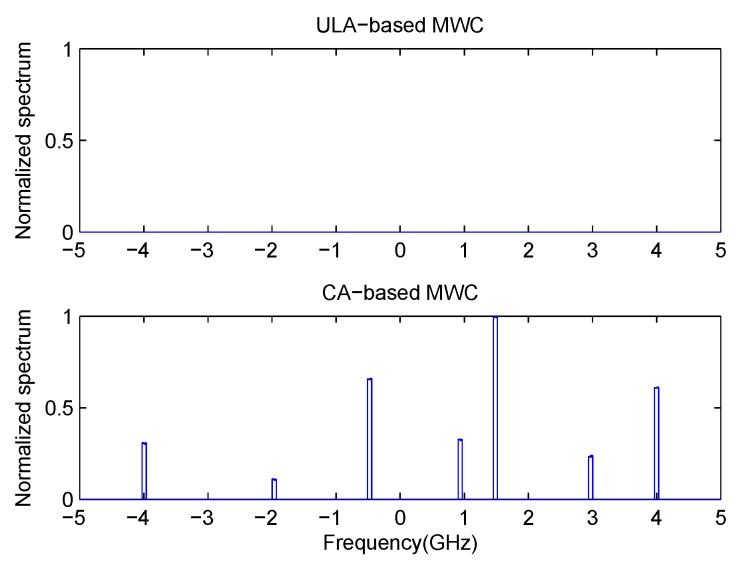
Detection performance in case of 7 transmissions with SNR = 10 dB.

In this experiment, we will examine the detection performance of our proposed method. Here, we set N=3 and M=2. So the first subarray has the sensor position [0,3,6,9]d, and the second subarray has the sensor position [0,2,4]d, with *d* taken as half of the wavelength corresponding to the Nyquist rate. The first sensor of these two subarrays are co-located. So the total number of sensors in our co-prime array is N+2M−1=6. Here, we compare our method with ULA-based MWC system. In the latter case, the number of physical sensors is also set as 6, which is the same as that of co-prime array. The Signal-to-noise rate (SNR) is set as 10 dB. Firstly, we assume that there are 3 transmissions with the carrier frequencies as [−1,1,1.5] GHz. In Figure 4, it can be seen that 3 transmissions can be detected clearly both in traditional ULA-based MWC system and our proposed CA-based MWC system. In addition, in Figure 4 (bottom), one transmission is a little higher than 1.5 GHz. This is caused by the randomness of p(t). For a specific run of CA-based MWC, it is possible that some elements in ${\left\{c_{l}\right\}}_{l=-L_{0}}^{L_{0}}$ are close to zero, which will degrade the estimating performance. But the probability is small. Then, we increase the number of transmissions to K=7 with the carrier frequencies as [−4,−2,−0.5,1,1.5,3,4] GHz. We can see from Figure 5 that our proposed method can detect all the transmissions successfully while traditional ULA-based MWC fails.

### 7.2. Sensing Accuracy

In the second experiment, we test the spectrum sensing accuracy of our proposed CA-based MWC system, compared with ULA-based MWC [10], traditional MWC [6] and non-compressive technique [9]. Because the number of uncorrelated transmissions which ULA-based MWC system can detect is no more than the number of physical sensors [9], we assume there are 3 transmissions with the carrier frequencies [−1,1,1.5] GHz. The SNR is ranged from 0 dB to 20 dB with step size 2 dB. The other simulation parameters are the same as those in the first experiment, e.g. N=3,M=2. So the total number of sensors used is N+2M−1=6. The number of sensors in ULA-based MWC system is also chosen as 6. In addition, the non-compressive method comes from [9] where we make the number of sampling channels equal to the decimator factor, namely $\left\lceil \frac{f_{\mathrm{Nyq}}}{f_{\mathrm{sys}}}\right\rceil =154$. Here, we define the spectrum sensing accuracy as

(62) $$\begin{array}{c} \varepsilon =\frac{1}{J}\sum_{j=1}^{J}\sqrt{\frac{1}{K}\sum_{i=1}^{K}\left[{\left({\hat{f}}_{i,j}^{l}-f_{i}^{l}\right)}^{2}+{\left({\hat{f}}_{i,j}^{u}-f_{i}^{u}\right)}^{2}\right]} \end{array}$$

where ${\hat{f}}_{i,j}^{l}$ and ${\hat{f}}_{i,j}^{u}$ are the estimated lower boundary and upper boundary of the *i*-th transmission in the *j*-th Monte Carlo simulation, respectively. $f_{i}^{l}$ and $f_{i}^{u}$ are the true lower boundary and upper boundary of *i*-th transmission. $f_{i}$ is the true carrier frequency of the *i*-th transmission. Here, 200 Monte Carlo simulations are used.

**Figure 6:**
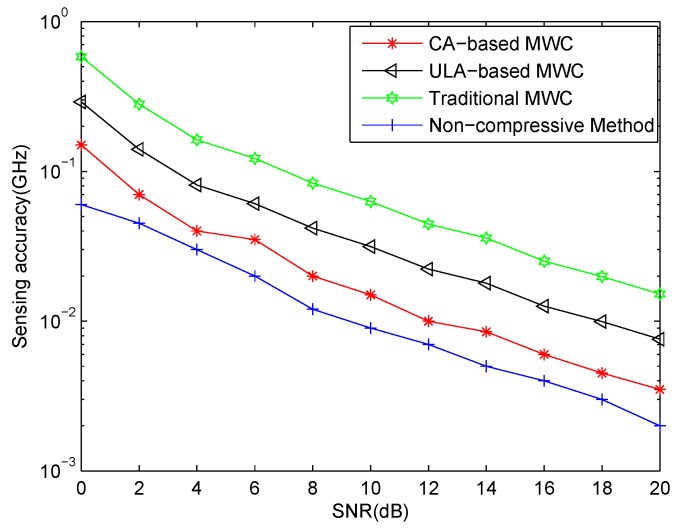
Sensing accuracy vs. SNR for 3 transmissions.

It can be seen that in Figure 6 non-compressive method has the best performance because it uses all Nyquist samples in signal processing. Array-based MWC methods, including CA-based MWC and ULA-based MWC, outperform traditional MWC method. This is on one hand due to the noise averaging in array-based system. On the other hand, array-based MWC methods have a two-step processing procedure, namely estimating carrier frequencies before recovering baseband transmissions, while conventional MWC method recovers the RF signal directly. Figure 6 also shows that our method has better performance than ULA-based MWC system. This is because in our method, a virtual ULA which has much larger aperture than ULA-based MWC system can be produced. A larger amount of sensors can increase the robustness to noise and allows it to handle a greater amount of sources. It can enhance CR performance.

**Figure 7:**
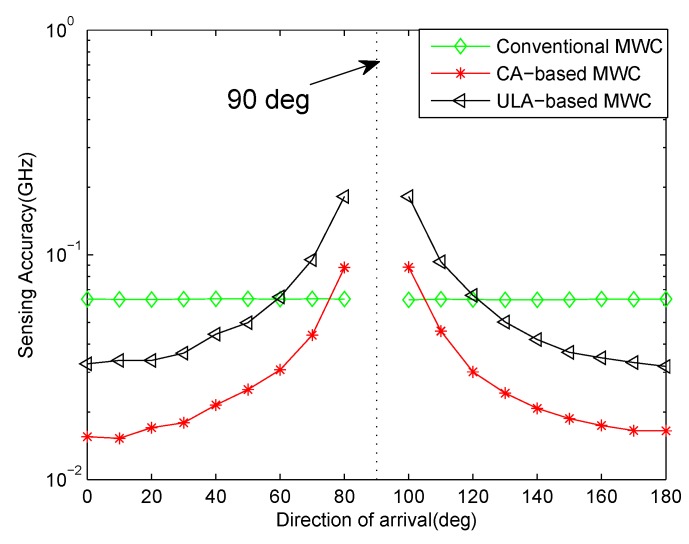
Sensing accuracy vs. direction-of-arrival for SNR = 10 dB.

Figure 6 is the result in the case of $\theta =0^{\circ }$. As analyzed in Section 4.1, the choice of θ can affect the performance of carrier frequency estimation, which will affect the sensing accuracy eventually. To investigate the sensing accuracy under different θ where $\theta \in \left[0^{\circ },90^{\circ }\right)\cup \left(90^{\circ },180^{\circ }\right]$, we fix SNR = 10 dB. For the interval $\left[0^{\circ },90^{\circ }\right)$, we choose θ from $0^{\circ }$ to $80^{\circ }$ with step size $10^{\circ }$. For the interval $\left(90^{\circ },180^{\circ }\right]$, we choose θ from $100^{\circ }$ to $180^{\circ }$ with step size $10^{\circ }$. The other parameters are the same as those of Figure 6. It can be seen from Figure 7 that the closer to $0^{\circ }$ or $180^{\circ }$ the impinging direction θ is, the smaller the estimation error is. Conversely, the closer to $90^{\circ }$, the larger the error is. And our proposed CA-based MWC method has a better performance than ULA-based MWC. In addition, we can see that the direction-of-arrival has no effect on conventional MWC method. This is because conventional MWC is a one-sensor, multichannel system, it does not use the time delays among different sensors which are caused by impinging angles. So conventional MWC method outperforms our proposed CA-based MWC when direction-of-arrival is close to $90^{\circ }$.

### 7.3. Parameter Choice Demonstration

**Figure 8:**
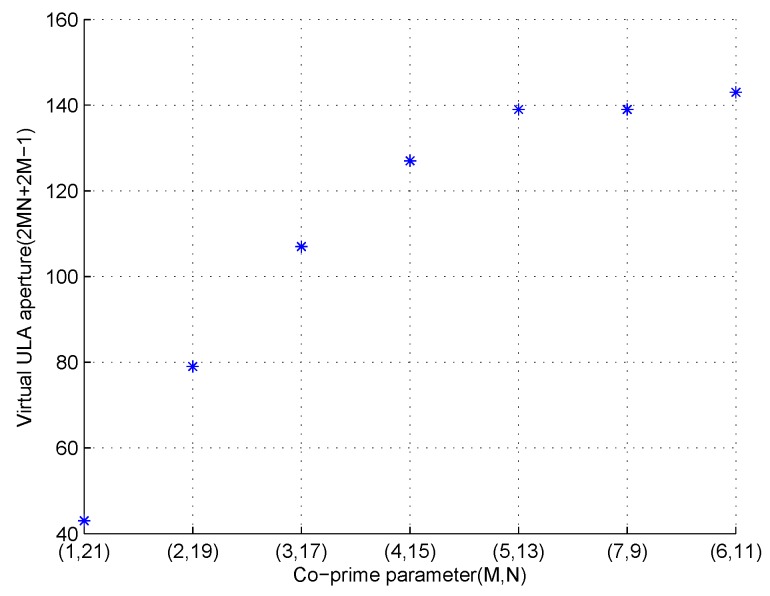
Virtual ULA apertures in different co-prime parameters.

In the third experiment, we demonstrate the co-prime parameter choice criterion in the fifth section. Unlike the above two experiments, we assume there are 22 physical sensors, namely Q=2M+N−1=22 where M<N. So we can list all the co-prime pairs (M,N) which satisfy the conditions, that’s (1,21),(2,19),(3,17),(4,15),(5,13),(6,11),(7,9). For each co-prime pair, the virtual ULA aperture is 2MN+2M−1. Figure 8 shows the virtual ULA apertures for different (M,N). It can be seen that the virtual ULA apertures vary in different co-prime parameters and the largest virtual ULA aperture appears when (M,N)=(6,11). This result coincides with our co-prime parameter choice criterion which says the largest virtual ULA aperture can be obtained when $\left(M,N\right)=\left(\frac{Q+2}{4},\frac{Q}{2}\right)=\left(6,11\right)$.

**Figure 9:**
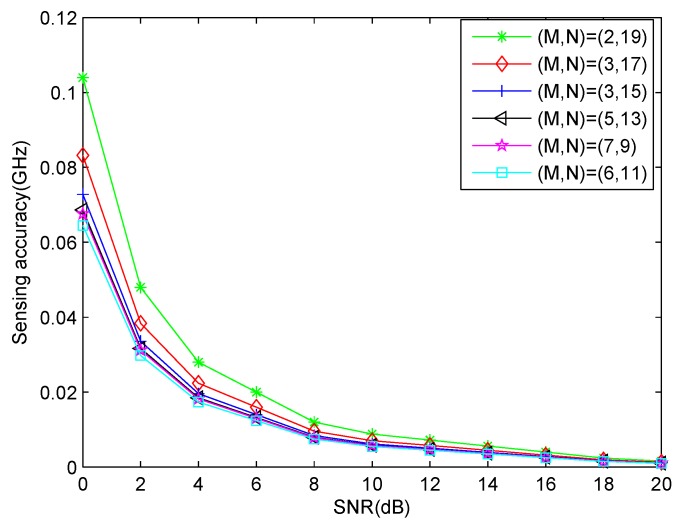
Sensing accuracy comparison among different co-prime parameters with Q=22.

Here, we also investigate the sensing performance under different co-prime parameters (M,N) for a fixed number of physical sensors Q=22. Because the co-prime parameter (1,21) has no sense in practice, we only compare the sensing accuracy under the co-prime parameters (2,19),(3,17),(4,15),(5,13),(7,9),(6,11). The other simulation parameters are the same as that of Section 7.2. It can been from Figure 9 that different co-prime parameters have different sensing performance. The larger the virtual ULA aperture is, the better the performance is.

### 7.4. Minimal System Sampling Rate Comparison

In the last experiment, we compare our proposed CA-based MWC with ULA-based MWC on minimal system sampling rate. As analyzed above, the system sampling rate $f_{\mathrm{sys}}$ is determined by the number of sampling channels *Q* and the low sampling rate $f_{s}$ of each channel, namely $f_{\mathrm{sys}}=Q\times f_{s}$. As shown in Section 3, our only requirement for $f_{s}$ is $f_{s}\geq f_{p}\geq B_{\max}$. Due to the existence of edge effect for signals, we make $f_{s}$ ($f_{p}$) a little larger than $B_{\max}$, that’s $f_{s}=f_{p}=1.3B_{\max}=65$ MHz. Obviously, $f_{s}$ is the same in both ULA-based MWC system and CA-based MWC system. The difference between these two systems is the required number of sampling channels *Q* for a fixed number of targets *K*. In the ULA-based MWC system, referring to [10], the minimal number of sampling channels required for *K* uncorrelated targets is K+1. So the corresponding minimal system sampling rate is $f_{\mathrm{sys}}=\left(K+1\right)f_{s}$. In our CA-based MWC system, based on Theorem 1, we can obtain the minimal number of sampling channels by solving the following optimization for a fixed *K*,

(63) $$\begin{array}{c} \min_{M,N}\;2M+N-1\\ s.t.\;MN+M>K,\gcd(M,N)=1,\\ N>M>0 \end{array}$$

**Figure 10:**
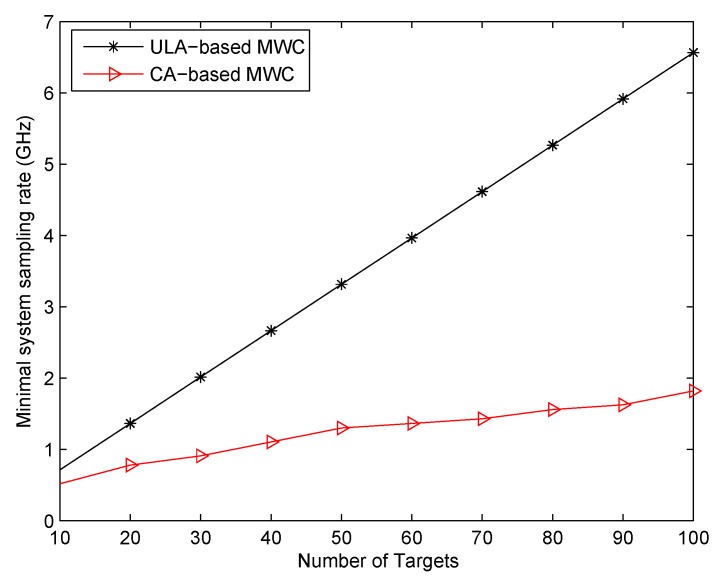
Comparison of system minimal sampling rate for different target numbers.

Denote the optimal solution as $\left(M^{*},N^{*}\right)$, then the minimal system sampling rate is $f_{\mathrm{sys}}=Q\times f_{s}=\left(2M^{*}+N^{*}-1\right)f_{s}$. Here, we make the number of targets from 10 to 100 with step size 10. It can be seen from Figure 10 that the system sampling rate of our proposed system is reduced largely compared to the ULA-based MWC system, especially when *K* is large.

## 8. Conclusions

In this paper, we used a co-prime array system for spectrum sensing of multiband signals. Each channel of our proposed CA-based MWC system is equivalent to one channel of traditional MWC system. The performance of our system outperforms traditional MWC system because the noise among all channels in our method is uncorrelated with each other, the noise can be averaged to increase the SNR. In addition, due to the incorporation of co-prime arrays, a virtual ULA which has a much larger aperture can be produced. Such virtual ULA can be used to detect more targets than traditional ULA-based MWC. Simulation results demonstrate the performance of our methods. In this paper, we mainly focus on the demonstration of feasibility of our proposed method which is based on computer experiment. Our next work is to design the whole hardware device of CA-based MWC system.

## Figures and Tables

**Table 1:** Comparisons of CA-based MWC, ULA-based MWC and MWC.

	CA-based MWC	ULA-based MWC	MWC
Periodic function	Same for all sensors	Same for all sensors	Different for all sensors
Number of channels	N+2M−1	N+2M−1	N+2M−1
Noise of each channel	Independent	Independent	Same
Processing domain	Coarray	Physical	Physical
Number of virtual elements	MN+M	×	×
Reconstruction	Power spectrum	Original signal	Original signal
